# Automatic AI-based contouring of prostate MRI for online adaptive radiotherapy

**DOI:** 10.1016/j.zemedi.2023.05.001

**Published:** 2023-05-30

**Authors:** Marcel Nachbar, Monica lo Russo, Cihan Gani, Simon Boeke, Daniel Wegener, Frank Paulsen, Daniel Zips, Thais Roque, Nikos Paragios, Daniela Thorwarth

**Affiliations:** a Section for Biomedical Physics, Department of Radiation Oncology, University Hospital and Medical Faculty, Eberhard Karls University of Tübingen, Tübingen, Germany; b Department of Radiation Oncology, University Hospital and Medical Faculty, Eberhard Karls University of Tübingen, Tübingen, Germany; c German Cancer Consortium (DKTK), partner site Tübingen; and German Cancer Research Center (DKFZ), Heidelberg, Germany; d Department of Radiation Oncology, Berlin Institute of Health, Charité - Universitätsmedizin Berlin, Corporate Member of Freie Universität Berlin, Humboldt-Universität zu Berlin, Berlin, Germany; e TheraPanacea, Paris, France; f CentraleSupelec, University of Paris-Saclay, Gif-sur-Yvette, France

**Keywords:** Deep learning, MR-Linac, Automatic annotations, MR-only, Adaptive radiotherapy

## Abstract

**Background and purpose:**

MR-guided radiotherapy (MRgRT) online plan adaptation accounts for tumor volume changes, interfraction motion and thus allows daily sparing of relevant organs at risk. Due to the high interfraction variability of bladder and rectum, patients with tumors in the pelvic region may strongly benefit from adaptive MRgRT. Currently, fast automatic annotation of anatomical structures is not available within the online MRgRT workflow. Therefore, the aim of this study was to train and validate a fast, accurate deep learning model for automatic MRI segmentation at the MR-Linac for future implementation in a clinical MRgRT workflow.

**Materials and methods:**

For a total of 47 patients, T2w MRI data were acquired on a 1.5 T MR-Linac (Unity, Elekta) on five different days. Prostate, seminal vesicles, rectum, anal canal, bladder, penile bulb, body and bony structures were manually annotated. These training data consisting of 232 data sets in total was used for the generation of a deep learning based autocontouring model and validated on 20 unseen T2w-MRIs. For quantitative evaluation the validation set was contoured by a radiation oncologist as gold standard contours (GSC) and compared in MATLAB to the automatic contours (AIC). For the evaluation, dice similarity coefficients (DSC), and 95% Hausdorff distances (95% HD), added path length (APL) and surface DSC (sDSC) were calculated in a caudal-cranial window of ± 4 cm with respect to the prostate ends. For qualitative evaluation, five radiation oncologists scored the AIC on the possible usage within an online adaptive workflow as follows: (1) no modifications needed, (2) minor adjustments needed, (3) major adjustments/ multiple minor adjustments needed, (4) not usable.

**Results:**

The quantitative evaluation revealed a maximum median 95% HD of 6.9 mm for the rectum and minimum median 95% HD of 2.7 mm for the bladder. Maximal and minimal median DSC were detected for bladder with 0.97 and for penile bulb with 0.73, respectively. Using a tolerance level of 3 mm, the highest and lowest sDSC were determined for rectum (0.94) and anal canal (0.68), respectively.

Qualitative evaluation resulted in a mean score of 1.2 for AICs over all organs and patients across all expert ratings. For the different autocontoured structures, the highest mean score of 1.0 was observed for anal canal, sacrum, femur left and right, and pelvis left, whereas for prostate the lowest mean score of 2.0 was detected. In total, 80% of the contours were rated be clinically acceptable, 16% to require minor and 4% major adjustments for online adaptive MRgRT.

**Conclusion:**

In this study, an AI-based autocontouring was successfully trained for online adaptive MR-guided radiotherapy on the 1.5 T MR-Linac system. The developed model can automatically generate contours accepted by physicians (80%) or only with the need of minor corrections (16%) for the irradiation of primary prostate on the clinically employed sequences.

## 1 Introduction

Online adaptive MR-guided radiotherapy (MRgRT) is now state of the art for a multitude of clinical entities, investigating the potential of hypofractionation, dose escalation on functional subvolumes, as well as reduction of margins for the potential sparing of organs at risk (OARs) [1], [2], [3], [4]. Even though a broad spectrum of different tumor sites is treated on MR-Linac systems, a primary focus is on prostate cancer due to the additional soft-tissue contrast and necessity of online adaptation due to movement and deformation of the prostate and seminal vesicles in dependency of bladder and rectum volume changes [5]. Within the current clinical workflow of online adaptive MRgRT, the most time consuming factor is a new daily accurate and consistent annotation of structures [6].This online workflow step not only limits the number of patients being treated, but also introduces time delays which can result in intrafractional motion of the relevant structures as reported by de Muinck Keizer et al. [7].

The clinical workflow of the 1.5 T MR-Linac system so far only supports a deformable registration from a primary CT or MRI to the daily image. This deformable registration however is limited due to deformations in the pelvic region and necessity of a baseline image. The results, as shown by Christiansen et al. [8] are strongly dependent of the baseline image type as well as to volume differences for structures like bladder and rectum. In order to improve the automatic annotation on MRI images, Eppenhof et al. [9] proposed for the 1.5 T MR-Linac system a fast deformable registration based on CNN of the baseline verified contours to the daily MRI. For the 0.35 T MR-Linac system Kawula et al. [10] proposed a patient-specific transfer learning approach, in which a pretrained baseline 3D-Unet can be improved with the offline baseline MRIs for each patient. For a baseline image independent automatic annotation on T2w-MRIs Elguindi et al. presented an autosegmentation model [11] and Cha et al. [12] the respective clinical implementation, using transfer learning on the two-dimensional fully convolutional neural network DeepLabV3+.

However, baseline MRI simulations are not always available prior to treatment on the MR-Linac system and anatomical changes between fractions unforeseen. Therefore, a deformable registration from a baseline image to the daily MRI has an inherit uncertainty based on anatomical differences and a patient-specific retraining might only be possible during the fractionated treatments. Additionally, whereas both MR-Linac systems use the MRI for daily imaging, due to the differences in magnetic field strength the 0.35 T MR-Linac employs a steady-state free-precession (bSSFP) imaging sequence with a T2∗/T1 image contrast and the 1.5 T MR-linac a T2-weighted spin echo sequence. Therefore, the image characteristics differ substantially and no direct comparison between the MR-Linac systems is possible. Additionally, due to the split magnet design, the hardware and consequently imaging characteristics of the 1.5 T MR-Linac system differ towards standard diagnostic MRI scanners. Especially the fast clinical 2 min T2-weighted imaging sequences of the MR-Linac can not be compared to diagnostic imaging [13] and published models on diagnostic imaging might not be directly usable on the MR-Linac system. An additional difference to a conventional radiotherapy workflow in online adaptive MRgRT is the necessity of organs at risk and target volumes as well as electron density given structures for the generation of a bulk density synthetic CT [14]. Therefore, so far no MRI autocontouring model for the 1.5 T MR-Linac is available, which does not employ a deformable registration of a baseline image and supports all necessary structures for a complete online MRgRT workflow.

The aim of this study was to train and validate a fast, accurate deep learning based automatic segmentation on imaging data of the 1.5 T MR-Linac system for the prospective implementation in a clinical MRgRT workflow. Therefore, the model should be capable to annotate the necessary organs at risk, target and electron density structures needed for the treatment of primary prostate cancer.

## 2 Material and methods

### 2.1 Patient selection

Training data was generated from MRI data of 47 pelvis patients treated at the MR-Linac between October 2018 and March 2020. The dataset consisted of a total of 232 T2-weighted MRIs, whereof 74% were acquired with a fast acquisition sequence taking 2 min and 26% with sequences with increased contrast. Details of the sequences and patient numbers are shown in Table S1. For every MRI all necessary structures supporting a complete online adaptive workflow were defined. For an assignment of densities sacrum, pelvis left and right, femur left and right and the patient body were considered relevant. As necessary organs at risk anal canal, bladder, penile bulb and rectum were considered. As target volumes prostate and seminal vesicles were chosen as anatomical structures since they can be used for a generation of CTV and PTV [15]. All structures were manually annotated offline of the MRgRT treatment by experienced technologists and approved by radiation oncologists, both teams with several years of experience at the MR-Linac system.

For model testing, data of 20 patients treated at the MRI-Linac between March 2020 and July 2021 were randomly selected. Patient characteristics for this cohort are given in Table 1. For every patient, one 2 min T2-weighted MRI acquired during fractionated MRgRT was randomly chosen for evaluation (cf. Table S1).

**Table 1:** Patient characteristics (n = 20).

**Body weight [kg]** mean ± standard deviation (SD)	80 ± 9
**Prostate volume [ccm]** mean ± SD	65 ± 18
**Patient age [y]** mean ± SD	76 ± 6
**Hip prosthesis** yes/no	0/20
**Transurethral prostate resection (TURP)** yes/no	2/18
**Gleason score** 6/7a/7b/8/9	1/6/9/2/2

All patients included in this study were treated under a phase 2 feasibility trial (NCT04172753). The study was approved by the institutional review board of the medical faculty Tübingen (IRB 659/2017B01) and all patients have given written informed consent.

### 2.2 Model generation

Annotate ART-Plan (V1.8.3, TheraPanacea), a commercially available auto-segmentation software that harnesses ensemble learning like anatomy preserving deep neural networks, was retrained using the data described in Section 2.1. Anatomically preserving data augmentation was performed (mapping the data to 4 reference anatomies), generating 928 training samples. The 4 reference anatomies were full body CT scans, selected by clinicians to be representative of the target patients, including male and female with different body shapes. They were used as preprocessing to ease the learning task by normalizing the MRIs onto fixed references as well to determine the structures of interest being present and approximate location.

A full end-to-end ensemble neural networks approach involving three steps was deployed: (i) a localization algorithm using affine registration to reference anatomies (ii) combination of the predictions from the neural networks with hard clinical constraints to ensure anatomical consistency (iii) standard ensemble learning approach by averaging the predictions of the 4 networks. Multiple networks were trained using different reference anatomies as references.

The AI models used were 3-dimensional full organ convolutional neural networks from the UNet family, the model parameters were adjusted across different structures. The adopted solution share concept similarities with Vakalopoulou et al. [16].

Once the deep neural network for AI-based auto-contouring was trained, it was applied to 20 unseen cases of pelvis T2w MR imaging data for clinical evaluation.

### 2.3 Quantitative evaluation

For the quantitative evaluation, one experienced board-certified radiation oncologist annotated the MRI data of the test cohort manually, without prior structural information. These gold standard contours (GSC) were compared against the AI-based automatic contours (AIC) for anal canal, bladder, rectum, seminal vesicles, prostate, sacrum, pelvis left and right as well as femur left and right in a caudal-cranial window of ± 4 cm with respect to the GSC prostate borders. To assess differences between GSC and AIC, the dice similarity coefficient (DSC) and the 95% Hausdorff-Distance (95% HD) were evaluated. In addition, to assess quality and the potential of the obtained contours to decrease MRgRT treatment time, surface DSC (sDSC) and added path length (APL) were calculated with tolerances varying between 1 and 5 mm in steps of 1 mm as defined by Vaassen et al. [17]. All quantitative metrics were calculated in MATLAB (Matlab V.2021a).

### 2.4 Qualitative evaluation

Five board-certified radiation oncologists experienced in the MR-Linac workflow scored the AIC on a four point likert scale with respect to potential clinical usage within an online MRgRT workflow. The four point Likert scale evaluated the structures according to the following scheme: (1) No modifications needed, (2) minor adjustments needed, (3) major adjustments/ multiple minor adjustments needed, (4) not usable within online adaptive MRgRT. Prior to independent scoring, one meeting was held with an exemplary patient case for a consensus agreement on the priorization towards usability of structures within online adaptive MRgRT. Each observer scored independently and no intermediate results were shared prior to finalization of the evaluation. For limited prior knowledge of patient anatomical features, at least 6 months have passed between clinical treatment and scoring of the chosen patients.

## 3 Results

### 3.1 Quantitative evaluation

**Figure 1:**
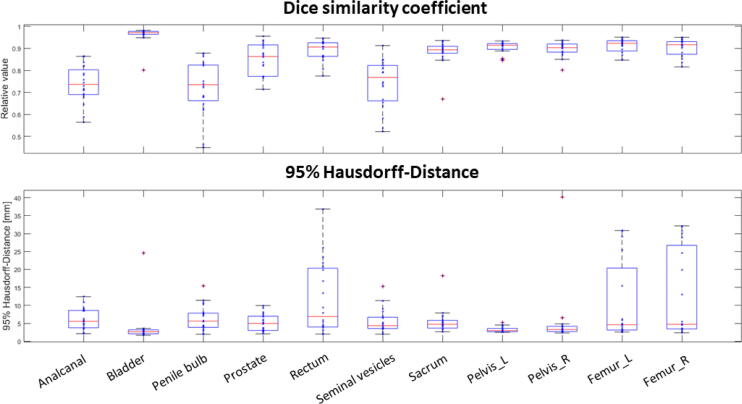
Quantitative results for the comparison of the automatic AI-based contours against gold standard manual contours. The top row depicts the dice similarity coefficient and the bottom row the 95% Hausdorff-Distance. Individual measurements are shown with blue asterixes. The median is shown in red and outliers are visualised with red crosses, if the distance towards median superseeds 1.5 × interquartile range.

**Figure 2:**
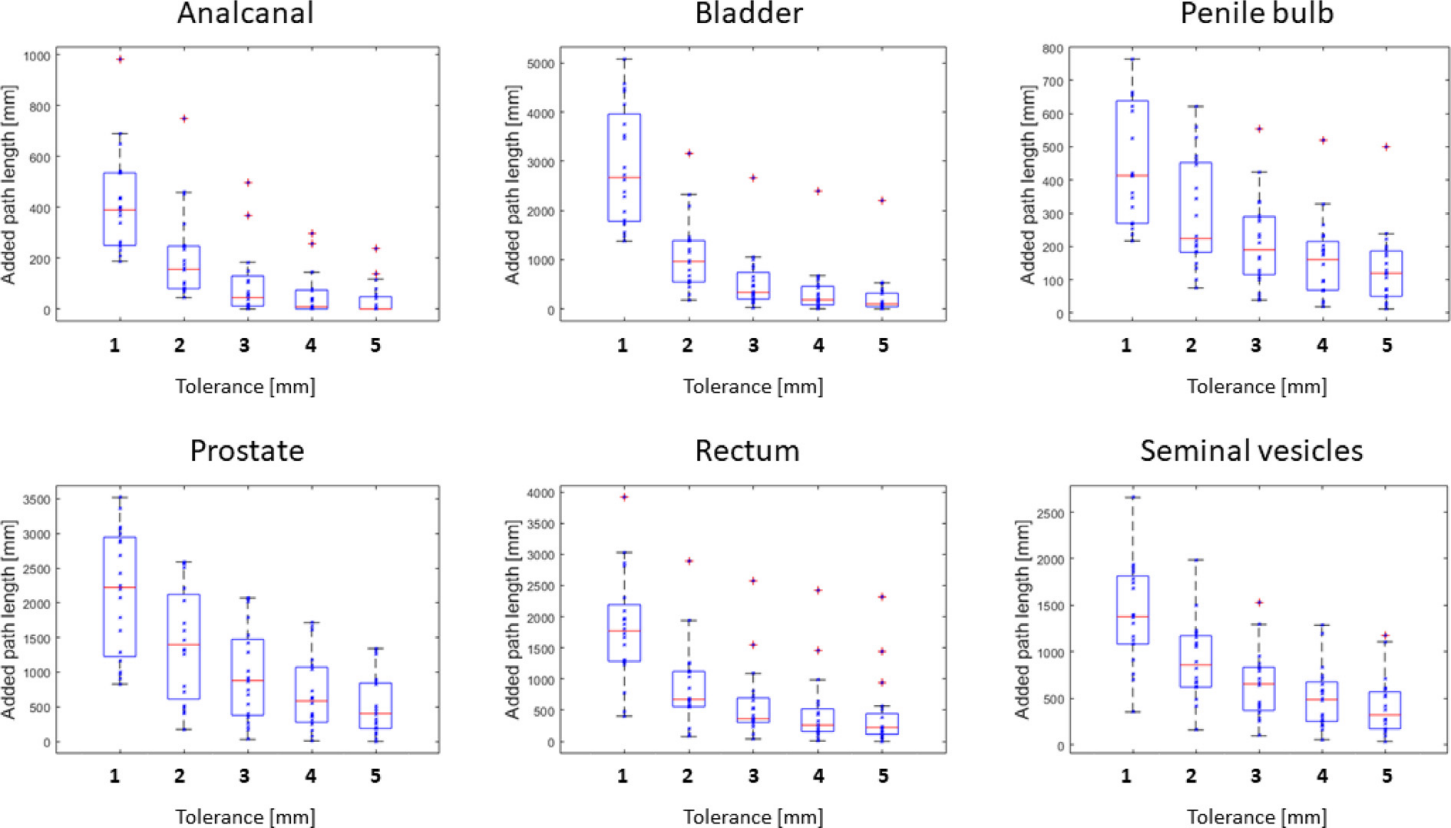
Analysis of the added path lengths for each OAR with the corresponding allowed tolerance between reference manual gold standard and tested automatic AI-based contours. Individual measurements are shown with blue asterixes. The median is shown in red and outliers are visualised with red crosses, if the distance towards median superseeds 1.5 × interquartile range.

For all patients, automatic AI-based segmentations were generated in a median [range] time of 152 s [121–198 s]. For OARs, the comparison of AIC and GSC showed a maximum median 95% HD of 6.9 mm for the rectum and and minimum median 95% HD of 2.7 mm for the bladder. The maximum 95% HD range in the test data set was found for rectum with 34.8 mm (min: 2.02 mm, max: 36.80 mm) and the smallest for the prostate with 7.9 mm (min: 2.08 mm, max: 9.95 mm). The maximal and minimal median DSC were detected for bladder with 0.97 and for penile bulb with 0.73 (cf. Fig. 1). Calculated APL showed a rapid decrease with increasing tolerances with the highest decrease of absolute APL over all OARs between 1 mm and 2 mm. For a tolerance of 3 mm the highest median APL was detected for the prostate with 876 mm and the smallest APL for the anal canal with 44 mm. For the same tolerance, the highest and lowest sDSC were determined for rectum (0.94) and anal canal (0.68), respectively. A visual respresentation of the APLs is presented in Fig. 2. A detailed overview of the results is summarized in Table 2.

**Table 2:** Results of the quantitative evaluation between AI-based contours (AIC) and gold standard (GSC) for organ at risk structures. Depicted are the median [range] results over all 20 evaluated patients for the 95% Hausdorff-Distance (95% HD), dice similarity coefficient (DSC), added path length (APL) and surface dice similarity coefficient (sDSC). For APL and sDSC the results are given in respect to the corresponding tolerances towards the GSC.

**Region of interest**			**Anal canal**	**Bladder**	**PenileBulb**	**Prostate**	**Rectum**	**SeminalVesicels**
95% HD [mm]			5.57 [2.14–12.41]	2.66 [1.70–24.60]	5.63 [1.97–15.40]	4.99 [2.08–9.95]	6.88 [2.02–36.80]	4.36 [2.01–15.32]
DSC			0.74 [0.56–0.86]	0.97 [0.80–0.98]	0.73 [0.45–0.88]	0.86 [0.71–0.96]	0.91 [0.77–0.95]	0.77 [0.52 −0.91]
APL [mm]	Tolerance	1 mm	390 [188–983]	2670 [1376–5078]	413 [217–764]	2223 [827–3520]	1770 [407–3912]	1376 [350–2658]
		2 mm	156 [44–749]	965 [182–3162]	224 [75–622]	1397 [173–2591]	676 [77–2898]	857 [159–1988]
		3 mm	44 [0–497]	339 [32–2661]	190 [39–555]	876 [28–2070]	364 [41–2576]	653 [97–1532]
		4 mm	9 [0–299]	186 [6–290]	160 [18–520]	585 [9–1715]	262 [11 2426]	487 [54–1286]
		5 mm	0 [0–239]	101 [0–2206]	119 [12–500]	402 [0–1339]	225 [0–2319]	321 [35–1180]
sDSC	Tolerance	1 mm	0.35 [0.06–0.66]	0.55 [0.32–0.70]	0.27 [0.06–0.60]	0.27 [0.04–0.67]	0.68 [0.47–0.86]	0.43 [0.21–0.73]
		2 mm	0.59 [0.28–0.92]	0.82 [0.63–0.94]	0.62 [0.29–0.92]	0.56 [0.21–0.92]	0.88 [0.73–0.96]	0.67 [0.39–0.91]
		3 mm	0.68 [0.44–0.97]	0.91 [0.69–0.98]	0.82 [0.45–0.97]	0.74 [0.47–0.97]	0.94 [0.82–0.99]	0.79 [0.57–0.96]
		4 mm	0.72 [0.51–0.98]	0.95 [0.71–00.99]	0.89 [0.54–0.98]	0.84 [0.59–0.99]	0.96 [0.86–1.00]	0.86 [0.67–0.99]
		5 mm	0.75 [0.52–0.98]	0.97 [0.73–0.99]	0.93 [0.60–0.99]	0.90 [0.72–1.00]	0.98 [0.88–1.00]	0.88 [0.75–0.99]

The structures necessary for electron density assignment (pelvis left/ right, femur left/ right and sacrum) showed a median DSC greater than 0.89 and a median 95% HD below 5 mm. The sDSC index showed comparable results between the paired structures femur left/ right and pelvis left/ right. For a tolerance of 3 mm the median sDSC ranged from 0.86 for pelvis right to 0.94 for femur left. A visual respresentation of the APLs is presented in Fig. S1 and a detailed summary of the results is given in Table S2.

### 3.2 Qualitative evaluation

**Figure 3:**
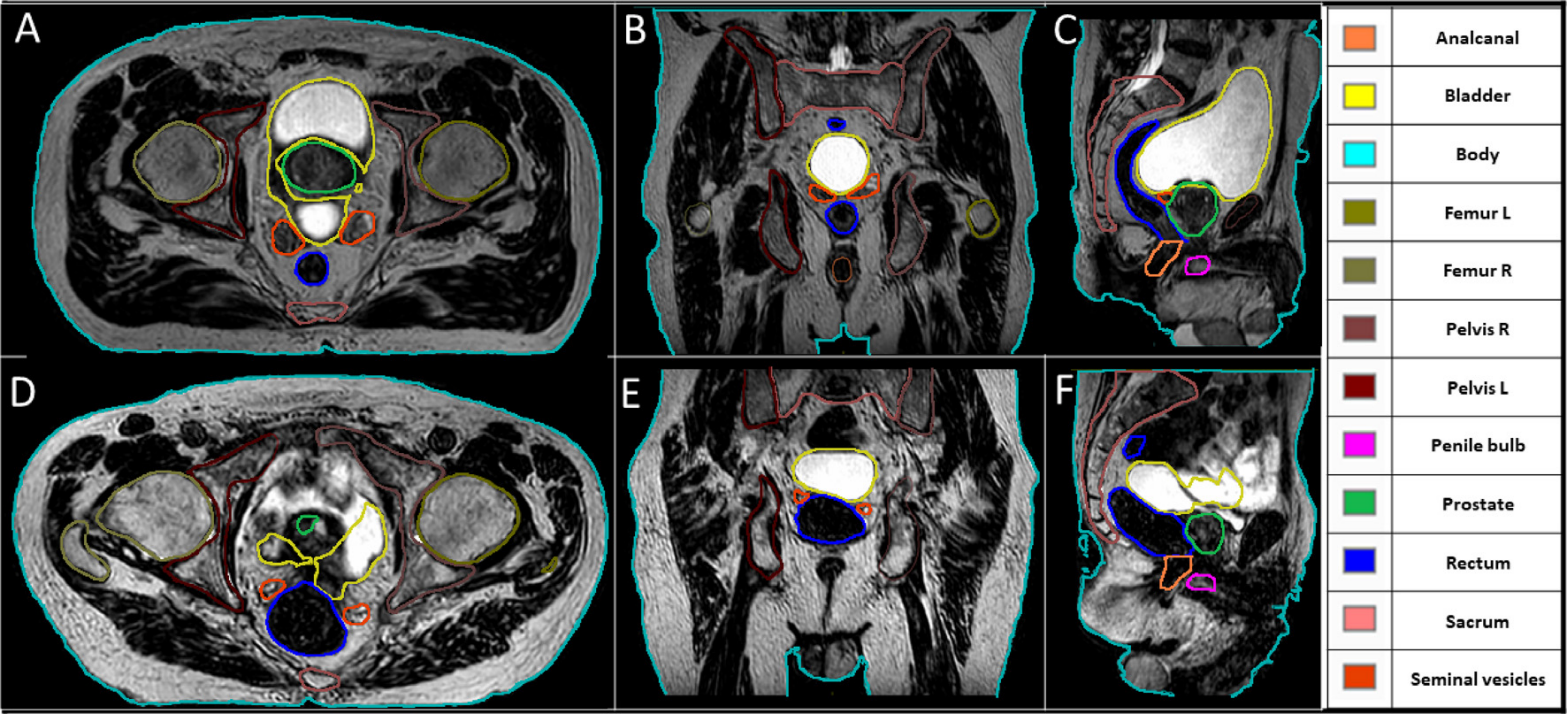
Visualization of the AI-based automatic segmentations for the best and worst mean results, based on the average physician-based score per patient over all structures. Therefore shown are patient number 18 (A-C) with an averaged score of 1.07 and patient number 14 (D-F) with a respective score of 1.58. Both patients are visualized in all three views at the center of gravity for the seminal vesicles.

Across all physicians, the AICs yielded a mean score of 1.2, for all organs and patients. Patient specific analysis revealed mean scores between 1.1 and 1.6 for patients 18 and 14, respectively. These two patients are visualised as representing best and worst case scenarios in Fig. 3.

**Figure 4:**
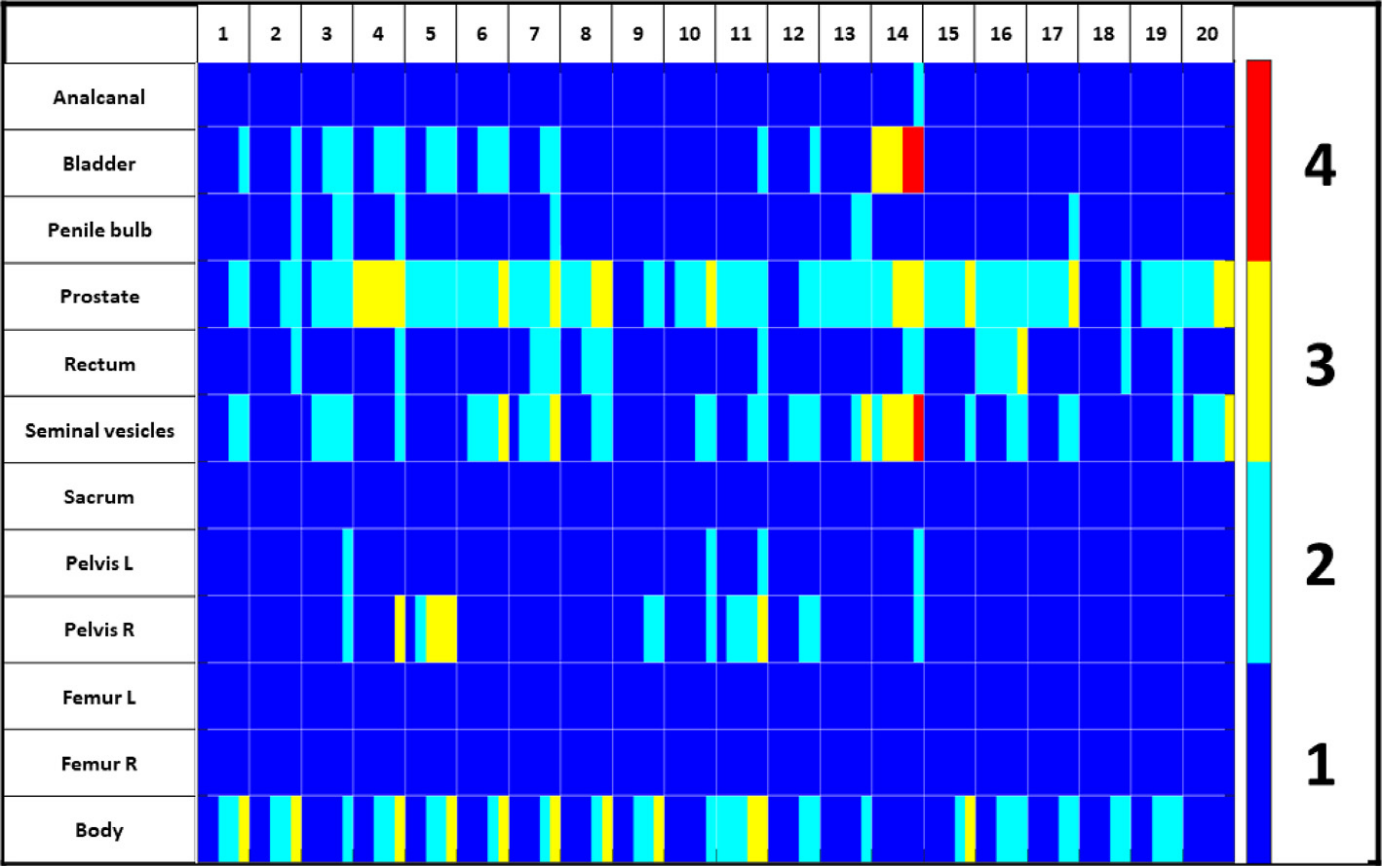
Heatmap of the qualitative physician-based scoring for the usage of the AI-based contours within an MRgRT-worklfow. For every patient and structure, the scores are visualised with a colorgrading. (1/ dark blue) No modifications needed, (2/ light blue) minor adjustments needed, (3/ yellow) major adjustments/ multiple minor adjustments needed, (4/ red) not usable. Within each box, the five physician-based scores were ordered from low (left) to high (right).

For the different autocontoured structures, the highest mean score was observed for anal canal, sacrum, femur left and right, and pelvis left which all scored an average of 1.0. The worst scoring was detected for the prostate with an average of 2.0. The prostate and seminal vesicles showed the highest standard deviation of scores between patients with 0.5. Additional details are presented in Table 3. A visualisation of scores for the different observers as a function of structure and patient is shown in Fig. 4 with a sorted colorgrading over the scored parameters. In total, 80% of all contours were scored to be clinically accpetable, whereas 16% were rated to require minor and 4% major changes for online adaptive MRgRT.

**Table 3:** Summary of the physician-based scoring on the usability of the AI-based autocontouring for MRgRT. For each patient and structure the shown value depicts the averaged scoring over all 5 physicians with the standard deviation between the observers. Scoring was done based on a 4 point Likert scale with (1) No modifications needed, (2) minor adjustments needed, (3) major adjustments/ multiple minor adjustments needed, (4) not usable.

**Structure/**	**Anal canal**	**Bladder**	**Penile Bulb**	**Prostate**	**Rectum**	**Seminal vessicle**	**Sacrum**	**Pelvis L**	**Pelvis R**	**Femur L**	**Femur R**	**Body**	**Mean patient score**
**Patient**													
**1**	1.0 ± 0.0	1.2 ± 0.4	1.0 ± 0.0	1.4 ± 0.5	1.0 ± 0.0	1.4 ± 0.5	1.0 ± 0.0	1.0 ± 0.0	1.0 ± 0.0	1.0 ± 0.0	1.0 ± 0.0	1.8 ± 0.7	1.15
**2**	1.0 ± 0.0	1.2 ± 0.4	1.2 ± 0.4	1.4 ± 0.5	1.2 ± 0.4	1.0 ± 0.0	1.0 ± 0.0	1.0 ± 0.0	1.0 ± 0.0	1.0 ± 0.0	1.0 ± 0.0	1.8 ± 0.7	1.15
**3**	1.0 ± 0.0	1.6 ± 0.5	1.4 ± 0.5	1.8 ± 0.4	1.0 ± 0.0	1.8 ± 0.4	1.0 ± 0.0	1.2 ± 0.4	1.2 ± 0.4	1.0 ± 0.0	1.0 ± 0.0	1.2 ± 0.4	1.27
**4**	1.0 ± 0.0	1.6 ± 0.5	1.2 ± 0.4	3.0 ± 0.0	1.2 ± 0.4	1.2 ± 0.4	1.0 ± 0.0	1.0 ± 0.0	1.4 ± 0.8	1.0 ± 0.0	1.0 ± 0.0	1.8 ± 0.7	1.37
**5**	1.0 ± 0.0	1.6 ± 0.5	1.0 ± 0.0	2.0 ± 0.0	1.0 ± 0.0	1.0 ± 0.0	1.0 ± 0.0	1.0 ± 0.0	2.4 ± 0.8	1.0 ± 0.0	1.0 ± 0.0	1.8 ± 0.7	1.32
**6**	1.0 ± 0.0	1.6 ± 0.5	1.0 ± 0.0	2.2 ± 0.4	1.0 ± 0.0	2.0 ± 0.6	1.0 ± 0.0	1.0 ± 0.0	1.0 ± 0.0	1.0 ± 0.0	1.0 ± 0.0	1.6 ± 0.8	1.28
**7**	1.0 ± 0.0	1.4 ± 0.5	1.2 ± 0.4	2.2 ± 0.4	1.6 ± 0.5	2.0 ± 0.6	1.0 ± 0.0	1.0 ± 0.0	1.0 ± 0.0	1.0 ± 0.0	1.0 ± 0.0	1.6 ± 0.8	1.33
**8**	1.0 ± 0.0	1.0 ± 0.0	1.0 ± 0.0	2.4 ± 0.5	1.6 ± 0.5	1.4 ± 0.5	1.0 ± 0.0	1.0 ± 0.0	1.0 ± 0.0	1.0 ± 0.0	1.0 ± 0.0	1.6 ± 0.8	1.25
**9**	1.0 ± 0.0	1.0 ± 0.0	1.0 ± 0.0	1.4 ± 0.5	1.0 ± 0.0	1.0 ± 0.0	1.0 ± 0.0	1.0 ± 0.0	1.4 ± 0.5	1.0 ± 0.0	1.0 ± 0.0	1.8 ± 0.7	1.13
**10**	1.0 ± 0.0	1.0 ± 0.0	1.0 ± 0.0	2.0 ± 0.6	1.0 ± 0.0	1.4 ± 0.5	1.0 ± 0.0	1.2 ± 0.4	1.2 ± 0.4	1.0 ± 0.0	1.0 ± 0.0	1.2 ± 0.4	1.17
**11**	1.0 ± 0.0	1.2 ± 0.4	1.0 ± 0.0	2.0 ± 0.0	1.2 ± 0.4	1.4 ± 0.5	1.0 ± 0.0	1.2 ± 0.4	2.0 ± 0.6	1.0 ± 0.0	1.0 ± 0.0	2.4 ± 0.5	1.37
**12**	1.0 ± 0.0	1.2 ± 0.4	1.0 ± 0.0	1.4 ± 0.5	1.0 ± 0.0	1.6 ± 0.5	1.0 ± 0.0	1.0 ± 0.0	1.4 ± 0.5	1.0 ± 0.0	1.0 ± 0.0	1.4 ± 0.5	1.17
**13**	1.0 ± 0.0	1.0 ± 0.0	1.4 ± 0.5	2.0 ± 0.0	1.0 ± 0.0	1.6 ± 0.8	1.0 ± 0.0	1.0 ± 0.0	1.0 ± 0.0	1.0 ± 0.0	1.0 ± 0.0	1.2 ± 0.4	1.18
**14**	1.2 ± 0.4	3.4 ± 0.5	1.0 ± 0.0	2.6 ± 0.5	1.4 ± 0.5	3.0 ± 0.6	1.0 ± 0.0	1.2 ± 0.4	1.2 ± 0.4	1.0 ± 0.0	1.0 ± 0.0	1.0 ± 0.0	1.58
**15**	1.0 ± 0.0	1.0 ± 0.0	1.0 ± 0.4	2.2 ± 0.4	1.0 ± 0.0	1.2 ± 0.4	1.0 ± 0.0	1.0 ± 0.0	1.0 ± 0.0	1.0 ± 0.0	1.0 ± 0.0	1.6 ± 0.8	1.17
**16**	1.0 ± 0.0	1.0 ± 0.0	1.0 ± 0.0	2.0 ± 0.0	2.2 ± 0.4	1.4 ± 0.5	1.0 ± 0.0	1.0 ± 0.0	1.0 ± 0.0	1.0 ± 0.0	1.0 ± 0.0	1.6 ± 0.5	1.27
**17**	1.0 ± 0.0	1.0 ± 0.0	1.2 ± 0.4	2.2 ± 0.4	1.0 ± 0.0	1.4 ± 0.5	1.0 ± 0.0	1.0 ± 0.0	1.0 ± 0.0	1.0 ± 0.0	1.0 ± 0.0	1.4 ± 0.5	1.18
**18**	1.0 ± 0.0	1.0 ± 0.0	1.0 ± 0.0	1.2 ± 0.4	1.2 ± 0.4	1.0 ± 0.0	1.0 ± 0.0	1.0 ± 0.0	1.0 ± 0.0	1.0 ± 0.0	1.0 ± 0.0	1.4 ± 0.5	1.07
**19**	1.0 ± 0.0	1.0 ± 0.0	1.0 ± 0.0	1.8 ± 0.4	1.2 ± 0.4	1.2 ± 0.4	1.0 ± 0.0	1.0 ± 0.0	1.0 ± 0.0	1.0 ± 0.0	1.0 ± 0.0	1.6 ± 0.5	1.15
**20**	1.0 ± 0.0	1.0 ± 0.0	1.0 ± 0.0	2.4 ± 0.5	1.0 ± 0.0	2.0 ± 0.6	1.0 ± 0.0	1.0 ± 0.0	1.0 ± 0.0	1.0 ± 0.0	1.0 ± 0.0	1.0 ± 0.0	1.20
**Mean**	1.0	1.3	1.1	2.0	1.2	1.5	1.0	1.0	1.2	1.0	1.0	1.5	1.24
**SD**	0.0	0.5	0.1	0.5	0.3	0.5	0.0	0.1	0.4	0.0	0.0	0.3	0.12

## 4 Discussion

In this study, we successfully trained and clinically evaluated a fast AI-based annotation model for anatomical T2-weighted MRIs of the 1.5 T MR-Linac system to support a workflow with reduced needed manual corrections during online adaptive MR-guided radiotherapy. We demonstrated that automatic annotation of all necessary structures on the daily adaptation MRIs of a 1.5 T MR-Linac system is possible. In a quantitative scoring the model was validated with an over all patients averaged median 95% HD and DSC of 5 mm and 0.83 for the organs at risk. For the necessary electron density structures, the gold standard was replicated with an average 95% HD and DSC of 4 mm and 0.91, respectively. These values were in addition verified in a physician-based scoring with a mean scoring over all patients of 1.3 and 1.1 for OARs and bony structures, respectively.

The qualitative evaluation using a subjective assessment by Likert scale showed a high overall acceptability of structures for the usage in an online MRgRT workflow. However, one individual patient, as visualised in Fig. 3, showed a strong decline in structure quality. For this patient case, a high signal was visible outside the bladder due to accumulation of fluid within the intestine. As this scenario is uncommon and was not part of the training dataset the automatically generated structures, especially near the liquid, could not be generated correctly. Therefore, such uncommon scenarios should be identified on bigger datasets either on visual inspection or based on the prediction uncertainty to include them within the model retraining. The deep learning literature on calibration proposes various methods to measure this calibration (Brier score, expected calibration error among others) and to calibrate networks, usually through temperature scaling, ensemble learning or Bayesian neural networks [18], [19], [20]. The generated training dataset is with 232 MRIs comparable in size to imaging datasets used within segmentation challenges [21], but due to multiple MRIs per patient, not all data samples can be considered independent. Therefore for further improvement, retraining of the model with more annotated data must be investigated. However, due to this uncertainty towards unseen patient anatomical configurations, an autonom patient treatment as proposed by Künzel et al. [15], [22] still needs the supervision of a physican. This could possibly be supplemented by extensive automatic quality assurances of structures similar to sanity checks in current synthetic CT software tools [23].

The quantitative evaluation showed a median DSC of 0.97, 0.86 and 0.91 for bladder, prostate and rectum, respectively. This was comparable to the published data from Kawula et al. [10], who reported values of 0.93 for bladder and 0.90 for rectum with an 3D U-Net-based automatic segmentation on MRIs of the 0.35 T MR-Linac system. To date, no complete solutions for a segmentation on MRIs are published which would allow a direct usage within online MRgRT due to various constellations of MR-Linac systems. Savenije et al. [24] published a deep learning based autosegmentation, annotating the femoral heads, bladder and rectum with a DSC of 0.97, 0.96 and 0.86. However, the proposed network is based on mDixon sequences, which are neither supported on the current MR-system, nor clinical standard within an online adaptive workflow [25]. In contrast, Elguindi et al. [11] showed based on axial diagnostic T2w-sequences, with their proposed DeepLabV3+ results comparable to ours with a DSC of 0.93 (bladder), 0.83 (prostate and seminal vesicles), 0.74 (penile bulb) and 0.82 (rectum). However, the proposed model does not support the necessary structures for an electron density assignment and is based on high quality T2-weighted axial MRIs, which are superior to MRIs from the 1.5 T MR-Linac system. In addition, the authors do not give an additional information about the needed time for an automatic annotation.

This model has been trained and validated only on the imaging quality of the 1.5 T MR-Linac system with differences in quality towards diagnostic MRIs [13]. The testing has only been analysed on the fast MRIs within the online MRgRT workflow and therefore is based on less contrast between structures, which increases anatomical uncertainites. However, the MRI of the hybrid 1.5 T MR-Linac system are commissioned based on the same protocols and tolerances, follow published quality assurances procedures and are limited in the supported sequences for comparable imaging between centers [26], [27], [28]. Therefore, the translatablility of the developed system between sites should be high but would still need to be validated in future studies.

A potential drawback of this model might be due to the inclusion of three different MRI-sequence types within the training data. Whereas this increases robustness, it also introduces intra-training variation of contrasts. Therefore, in further studies the two additional MRI-sequences should be evaluated on their effect within the training as well as the potential of increasing training data by including diagnostic imaging data.

An automatic annotation of organs at risk as well as target structures is not only important for reducing workflow time, but can also serve as a basis for dose accumulation. Whereas deformable image registration can be executed without prior delineations guiding a structure along similar structures increases the quality of deformation and thus the accuracy of dose accumulation, even if the structure is not perfectly aligned.

Another potential of automatic delineation on the clinical MRIs is a standardized retrospective evaluation on a high number of patients as exact manual contouring on the MRIs is very time-consuming. This would allow for a better investigation of inter- and intrafractional movement of substructures as well as the replication of daily applied organ doses during therapy in order to generate more accurate normal tissue complication probabilities models [29].

The analysis of this study is only based on a quantitative and qualitative evaluation, no dosimetric evaluation of differences between the GSC and AIC was performed as this is very depending on the chosen margin recipe as well as on the treatment planning strategy. However, additional quantitative metrics as the APL and sDSC were calculated based on the publication of Vaassen et al. [17] with different tolerances between the physician and automatic annotation. Based on this, the structure specific overlap information in dependency of the evaluated tolerance could be used for margin definitions in planning organ at risk volumes, supporting a fast treatment with online MRgRT [30].

In addition, APL assessment allows an estimation of required additional contouring time in order to correct AI-based contours. However, to calculate a realistic time estimation a calibration curve of APL in correlation to contour correction time in the circumstances of online adaptive therapy and corresponding voxel dimensions must be recorded [17]. Whereas the deep learning based contouring is faster than manual contouring, with a median annotation time of 152 s, this time difference must be accounted for in real-time adaptive therapy.

The quantitative evaluation was limited to a caudal-cranial window of 4 cm with respect to the primary prostate, as this is the area in which the contours will be adapted during online adaptive radiotherapy. However, in an additional evaluation differences between the structures are visible at the cranial border of the rectum towards the sigmoid S2, limiting its current usage for abdominal treatment sites.

Whereas for most OARs the proposed model showed comparable results to other automatic contouring software, it is not yet in the range of the intraobserver variability for the target structures such as prostate gland as shown by Montagne et al., who detected on high quality 3 T T2-weighted images a DSC of 0.92 between seven observers for the whole prostate gland [31]. To increase the quality for MRgRT, future possible improvements might be achieved by improving the MRI quality for better distinction between structures. An additional opportunity is a patient specific retraining of the autocontouring based on the first fraction as published by Fransson et al. [32]. However, this concept would be highly time-consuming and depends on a lot of experience on site. A third option would be to screen every automatically annotated patient during the daily treatment and trigger a retraining loop, if the models prediction surpasses an uncertainty threshold, or if the contours were deemed not acceptable by the physician. This would allow for a generalised model increasing in quality concurrent with the number of treated patients.

Within this work, the necessary structures were defined based on prescribed organs at risk in the most recently published clinical trials for primary prostate to support a full clinical workflow [33], [34]. However, future work should include additional structures such as the urethra, for which toxicity models have been recently published [35] as well as explore other tumor sites such as head and neck.

Based on this study, treatment sites could directly employ the model on a first simulation scan for initial reference structures. After the validation of a physician and physicist for OARs and electron density given structures, the contours can be used directly for the generation of a reference treatment plan with a bulk density approach as proposed by Coric et al. [36].

## 5 Conclusion

In this study an AI-based autocontouring was successfully trained for online adaptive MR-guided radiotherapy on the 1.5 T MR-Linac system for the pelvic anatomy. The developed model can automatically generate contours accepted by physician (80%) or only with the need of minor corrections (16%) for the irradiation of primary prostate on the clinically employed sequences. This promises a quicker annotation on the T2-weighted images, including all necessary structures for a bulk density assignment, which can ultimately lead to higher patient throughput of MR-Linac systems. In a next step, the active integration of the autocontouring within the online adaptive workflow should be initiated and a constant improvement of the model by active integration of unknown patient anatomies considered.

## Declaration of Competing Interest

The authors declare the following financial interests/personal relationships which may be considered as potential competing interests: [The authors report institutional collaborations with TheraPanacea, Elekta, Philips, Kaiku and PTW Freiburg which provided technical and/or financial support. TheraPanacea developed the proposed model, but were not involved in the analysis and scoring of the testing dataset.]

## References

[1] Christiansen R.L., Dysager L., Hansen C.R., Jensen H.R., Schytte T., Nyborg C.J., Bertelsen A.S., Agergaard S.N., Mahmood F., Hansen S., Hansen O., Brink C., Bernchou U. (2022). Online adaptive radiotherapy potentially reduces toxicity for high-risk prostate cancer treatment. Radiother Oncol.

[2] Nachbar M., Mönnich D., Boeke S., Gani C., Weidner N., Heinrich V., Lo Russo M., Livi L., Winter J., Tsitsekidis S., Dohm O., Thorwarth D., Zips D., De-Colle C. (2019). Partial breast irradiation with the 1.5 T MR-Linac: First patient treatment and analysis of electron return and stream effects. Radiother Oncol.

[3] Thorwarth D., Ege M., Nachbar M., Mönnich D., Gani C., Zips D., Boeke S. (2020). Quantitative magnetic resonance imaging on hybrid magnetic resonance linear accelerators: Perspective on technical and clinical validation. Phys Imag Radiat Oncol.

[4] Gani C., Lo Russo M., Boeke S., Wegener D., Gatidis S., Butzer S., Boldt J., Mönnich D., Thorwarth D., Nikolaou K., Zips D., Nachbar M. (2021). A novel approach for radiotherapy dose escalation in rectal cancer using online MR-guidance and rectal ultrasound gel filling - Rationale and first in human. Radiother Oncol.

[5] Muinck Keizer D., Willigenburg T., der Voort van Zyp J., Raaymakers B., Lagendijk J., Boer J. (2021). Seminal vesicle intrafraction motion during the delivery of radiotherapy sessions on a 1.5 T MR-Linac. Radiother Oncol.

[6] Willigenburg T, Muinck Keizer DM de, Peters M, Claes A, Lagendijk JJW, Boer HCJ de, Voort van Zyp JRN van der. Evaluation of daily online contour adaptation by radiation therapists for prostate cancer treatment on an MRI-guided linear accelerator. Clin Translat Radiat Onco 2021;27:50–56. https://doi.org/10.1016/j.ctro.2021.01.002. 10.1016/j.ctro.2021.01.002 PMC7822780 33532630

[7] de Muinck Keizer D., Kerkmeijer L., Willigenburg T., van Lier A., den Hartogh M., van der Voort van Zyp J., de Groot-van Breugel E., Raaymakers B., Lagendijk J., de Boer J. (2020). Prostate intrafraction motion during the preparation and delivery of MR-guided radiotherapy sessions on a 1.5T MR-Linac. Radiother Oncol.

[8] Christiansen R.L., Dysager L., Bertelsen A.S., Hansen O., Brink C., Bernchou U. (2020). Accuracy of automatic deformable structure propagation for high-field MRI guided prostate radiotherapy. Radiat Oncol.

[9] Eppenhof KAJ, Maspero M, Savenije MHF, Boer JCJ de, Voort van Zyp JRN van der, Raaymakers BW, Raaijmakers AJE, Veta M, Berg CAT van den, Pluim JPW. Fast contour propagation for MR-guided prostate radiotherapy using convolutional neural networks. Med Phys 2020;47:1238–1248. https://doi.org/10.1002/mp.13994. 10.1002/mp.13994 PMC7079098 31876300

[10] Kawula M., Hadi I., Nierer L., Vagni M., Cusumano D., Boldrini L., Placidi L., Corradini S., Belka C., Landry G., Kurz C. (2023). Patient-specific transfer learning for auto-segmentation in adaptive 0.35 T MRgRT of prostate cancer: a bi-centric evaluation. Med Phys.

[11] Elguindi S., Zelefsky M.J., Jiang J., Veeraraghavan H., Deasy J.O., Hunt M.A., Tyagi N. (2019). Deep learning-based auto-segmentation of targets and organs-at-risk for magnetic resonance imaging only planning of prostate radiotherapy. Phys Imag Radiat Oncol.

[12] Cha E., Elguindi S., Onochie I., Gorovets D., Deasy J.O., Zelefsky M., Gillespie E.F. (2021). Clinical implementation of deep learning contour autosegmentation for prostate radiotherapy. Radiother Oncol.

[13] Almansour H., Afat S., Fritz V., Schick F., Nachbar M., Thorwarth D., Zips D., Müller A.-C., Nikolaou K., Othman A.E. (2021). Prospective image quality and lesion assessment in the setting of MR-guided radiation therapy of prostate cancer on an MR-linac at 1.5 T: a comparison to a standard 3 T MRI. Cancers.

[14] Winkel D., Bol G.H., Kroon P.S., van Asselen B., Hackett S.S., Werensteijn-Honingh A.M., Intven M.P.W., Eppinga W.S.C., Tijssen R.H.N., Kerkmeijer L.G.W., de Boer H.C.J., Mook S., Meijer G.J., Hes J., Willemsen-Bosman M., de Groot-van Breugel E.N., Jürgenliemk-Schulz I.M., Raaymakers B.W. (2019). Adaptive radiotherapy: The Elekta Unity MR-linac concept. Clin Transl Radiat Oncol.

[15] Künzel L.A., Nachbar M., Hagmüller M., Gani C., Boeke S., Zips D., Thorwarth D. (2021). First experience of autonomous, un-supervised treatment planning integrated in adaptive MR-guided radiotherapy and delivered to a patient with prostate cancer. Radiother Oncol.

[16] Vakalopoulou M, Chassagnon G, Bus N, Marini R, Zacharaki E, Revel M-P, Paragios N. Atlasnet: Multi-atlas non-linear deep networks for medical image segmentation. 218; 2018.

[17] Vaassen F., Hazelaar C., Vaniqui A., Gooding M., van der Heyden B., Canters R. (2020). Elmpt W van Evaluation of measures for assessing time-saving of automatic organ-at-risk segmentation in radiotherapy. Phys Imag Radiat Oncol.

[18] Guo C, Pleiss G, Sun Y, Weinberger KQ. On calibration of modern neural networks. In: Proceedings of the 34th International Conference on Machine Learning. Ed. by D Precup, Y W Teh. Vol. 70. Proceedings of Machine Learning Research. PMLR, June 2017. pp. 1321–1330.

[19] Gal Y, Ghahramani Z Dropout as a bayesian approximation: Representing model uncertainty in deep learning. In: International conference on machine learning. PMLR; 2016. pp. 1050–1059.

[20] Wenzel F., Snoek J., Tran D., Jenatton R., Larochelle H., Ranzato M., Hadsell R., Balcan M., Lin H. (2020).

[21] Antonelli M., Reinke A., Bakas S., Farahani K., Kopp-Schneider A., Landman B.A. (2022). The medical segmentation decathlon. Nat. Commun..

[22] Künzel L.A., Nachbar M., Hagmüller M., Gani C., Boeke S., Wegener D., Paulsen F., Zips D., Thorwarth D. (2022). Clinical evaluation of autonomous, unsupervised planning integrated in MR-guided radiotherapy for prostate cancer. Radiother Oncol.

[23] Tyagi N., Zelefsky M.J., Wibmer A., Zakian K., Burleson S., Happersett L., Halkola A., Kadbi M., Hunt M. (2020). Clinical experience and workflow challenges with magnetic resonance-only radiation therapy simulation and planning for prostate cancer. Phys Imag Radiat Oncol.

[24] Savenije MHF, Maspero M, Sikkes GG, Voort van Zyp JRN van der, T.J. Kotte AN, Bol GH, T. van den Berg CA. Clinical implementation of MRI-based organs-at-risk auto-segmentation with convolutional networks for prostate radiotherapy. Radiat Oncol 2020;15:104. https://doi.org/10.1186/s13014-020-01528-0. 10.1186/s13014-020-01528-0 PMC7216473 32393280

[25] Yang J., Vedam S., Lee B., Castillo P., Sobremonte A., Hughes N., Mohammedsaid M., Wang J., Choi S. (2021). Online adaptive planning for prostate stereotactic body radiotherapy using a 1.5 Tesla magnetic resonance imaging-guided linear accelerator. Physics and Imaging. Radiat Oncol.

[26] Kooreman E.S., van Houdt P.J., Nowee M.E., van Pelt V.W.J., Tijssen R.H.N., Paulson E.S., Gurney-Champion O.J., Wang J., Koetsveld F., van Buuren L.D., Ter Beek L.C., Heide U. (2019). A van der Feasibility and accuracy of quantitative imaging on a 1.5 T MR-linear accelerator. Radiother Oncol.

[27] Tijssen R.H., Philippens M.E., Paulson E.S., Glitzner M., Chugh B., Wetscherek A., Dubec M., Wang J., van der Heide U. (2019). A MRI commissioning of 1.5T MR-linac systems – a multi-institutional study. Radiother Oncol.

[28] Roberts D.A., Sandin C., Vesanen P.T., Lee H., Hanson I.M., Nill S., Perik T., Lim S.B., Vedam S., Yang J., Woodings S.W., Wolthaus J.W.H., Keller B., Budgell G., Chen X. (2021). Li X A Machine QA for the Elekta Unity system: A Report from the Elekta MR-linac consortium. Med Phys.

[29] Dréan G., Acosta O., Ospina J.D., Fargeas A., Lafond C., Corrégé G., Lagrange J.-L., Créhange G., Simon A., Haigron P. (2016). de Crevoisier R Identification of a rectal subregion highly predictive of rectal bleeding in prostate cancer IMRT. Radiother Oncol.

[30] Muren L.P., Karlsdottir Á., Kvinnsland Y., Wentzel-Larsen T. (2005). Dahl O Testing the new ICRU 62 ’Planning Organ at Risk Volume’ concept for the rectum. Radiother Oncol.

[31] Montagne S., Hamzaoui D., Allera A., Ezziane M., Luzurier A., Quint R., Kalai M., Ayache N., Delingette H. (2021). Renard-Penna R Challenge of prostate MRI segmentation on T2-weighted images: inter-observer variability and impact of prostate morphology. Insights Imag.

[32] Fransson S., Tilly D. (2022). Strand R Patient specific deep learning based segmentation for magnetic resonance guided prostate radiotherapy. Phys Imaging Radiat Oncol.

[33] Dearnaley D., Syndikus I., Sumo G., Bidmead M., Bloomfield D., Clark C. (2012). Conventional versus hypofractionated high-dose intensity-modulated radiotherapy for prostate cancer: preliminary safety results from the CHHiP randomised controlled trial. Lancet Oncol.

[34] Kerkmeijer LGW, Groen VH, Pos FJ, Haustermans K, Monninkhof EM, Smeenk RJ, Kunze-Busch M, Boer JCJ d, Zijp JvdVv, Vulpen Mv, Draulans C, Bergh Lvd, Isebaert S, Heide UAvd. Focal boost to the intraprostatic tumor in external beam radiotherapy for patients with localized prostate cancer: results from the FLAME randomized phase III trial. J Clin Oncol2021;39:787–796. https://doi.org/10.1200/jco.20.02873]. 10.1200/JCO.20.02873 33471548

[35] Leeman J.E., Chen Y.-H., Catalano P., Bredfeldt J., King M., Mouw K.W., D’Amico A.V., Orio P., Nguyen P.L., Martin N. (2022). Radiation dose to the intraprostatic urethra correlates strongly with urinary toxicity after prostate stereotactic body radiation therapy: A combined analysis of 23 prospective clinical trials. Int J Radiat Oncol*Biol*Phys.

[36] Coric I., Shreshtha K., Roque T., Paragios N., Gani C., Zips D., Thorwarth D., Nachbar M. (2022). Dosimetric evaluation of dose calculation uncertainties for MR-only approaches in prostate MR-guided radiotherapy. Front Phys.

