# Supplementary material for: Automatic AI-based contouring of prostate MRI for online adaptive radiotherapy
Source: Z Med Phys. 2023 May 30;34(2):197–207. doi: 10.1016/j.zemedi.2023.05.001 (PMC11156783; doi:10.1016/j.zemedi.2023.05.001)
Supplement: Supplementary data 1 [file mmc1.pdf]

Supplementary Materials

Table S1: Summary of included patients in training and testing data in correspondence to the employed sequences. The total number of independent patients were 47. The number of independent patients within the datasets corresponds to the number of independent patients within each MRI-sequence. The sequence T2-3D pss stands for T2-3D pseudo-steady-state.

Sequence	T2-2min	T2-3D	T2-3D pss
FOV (AP × RL × FH) [mm]	400 × 400 × 300	400 × 448 × 250	400 × 448 × 250
Acquisition voxel size [mm]	1.5 × 1.5 × 2	1.2 × 1.2 × 1.2	1.2 × 1.2 × 1.2
Reconstructed voxel size [mm]	0.83 × 0.83 × 1	0.52 × 0.52 × 1.2	0.52 × 0.52 × 0.6
Flip angle [°]	90	90	90
TE [ms]	278	82	168
TR [ms]	1535	1300	1300
Scantime [min]	01:57	6:51	5:30
MRIs in training data	171	39	22
MRIs in testing data	20	0	0

Table S2: Results of the quantitative evaluation between AI-based contours and gold standard for structures needed for electron density assignments. Depicted are the median [range] results overall 20 evaluated patients for the 95% Hausdorff-Distance (95% HD), dice similarity coefficient (DSC), added path length (APL) and surface dice similarity coefficient (sDSC). For APL and sDSC the results are given in respect to the corresponding tolerances towards the GSC.

Region of interest			Sacrum	Pelvis_L	Pelvis_R	Femur_L	Femur_R
95% HD [mm]			4.75 [2.64-18.26]	2.51 [2.51-5.23]	3.26 [2.33-40.17]	4.62 [2.59-30.82]	4.73 [2.36-32.14]
DSC			0.89 [0.67-0.94]	0.91 [0.85-0.93]	0.90 [0.80-0.94]	0.92 [0.85-0.95]	0.92 [0.82-0.95]
APL [mm]	Tolerance	1 mm	2907 [821-4883]	7876 [5826-10178]	9298 [5843-13084]	5686 [3175-7171]	5925 [2121-9130]
		2 mm	1283 [246-2633]	3649 [2106-6063]	4214 [2180-7785]	1323 [626-3860]	1645 [219-4556]
		3 mm	528 [67-1408]	1583 [840-3789]	2231 [1193-5552]	416 [168-2432]	479 [64-2340]
		4 mm	267 [33-853]	897 [331-2552]	1401 [703-5112]	190 [79-1155]	224 [28-1313]
		5 mm	191 [23-648]	539 [168-1677]	1010 [411-4890]	123 [51 453]	157 [26-890]
sDSC	Tolerance	1 mm	0.37 [0.20-0.49]	0.41 [0.23-0.55]	0.39 [0.17-0.54]	0.41 [0.34-0.59]	0.42 [0.27-0.62]
		2 mm	0.70 [0.49-0.81]	0.75 [0.49-0.83]	0.71 [0.41-0.84]	0.80 [0.61-0.90]	0.78 [0.61-0.90]
		3 mm	0.86 [0.71-0.95]	0.88 [0.69-0.94]	0.86 [0.63-0.92]	0.94 [0.75-0.97]	0.92 [0.80-0.97]
		4 mm	0.92 [0.80-0.98]	0.94 [0.79-0.97]	0.92 [0.77-0.96]	0.97 [0.87-0.99]	0.97 [0.91-0.99]
		5 mm	0.95 [0.83-0.99]	0.96 [0.86-0.99]	0.95 [0.84-0.98]	0.99 [0.94-1.00]	0.98 [0.96-0.99]

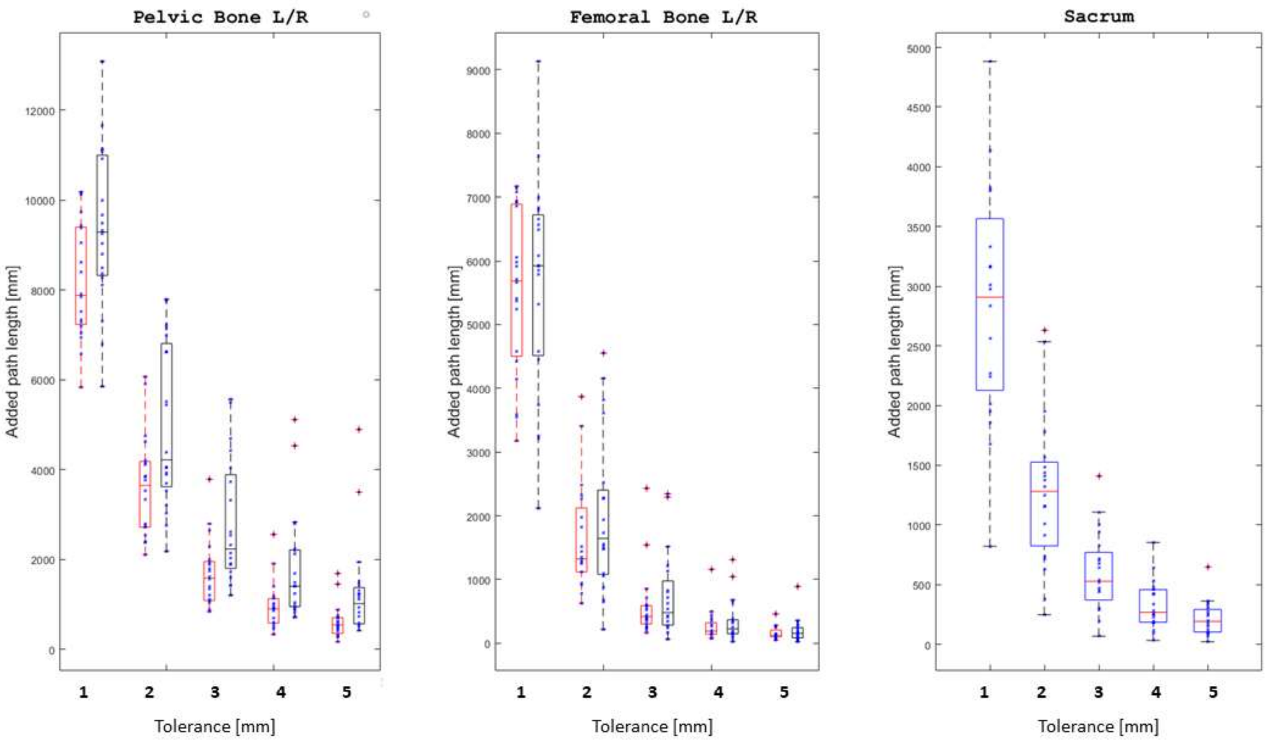

Figure S1: Analysis of the added path lengths for each structure with the corresponding allowed tolerance between reference manual gold standard and tested automatic AI-based contours. Individual measurements are shown with blue asterixes. The median is shown in red and outliers are visualised with red crosses, if the distance towards median superseeds 1.5 × interquartile range. For pelvis bone and femoral bone the corresponding results for left and right side is visualised in red and blue.

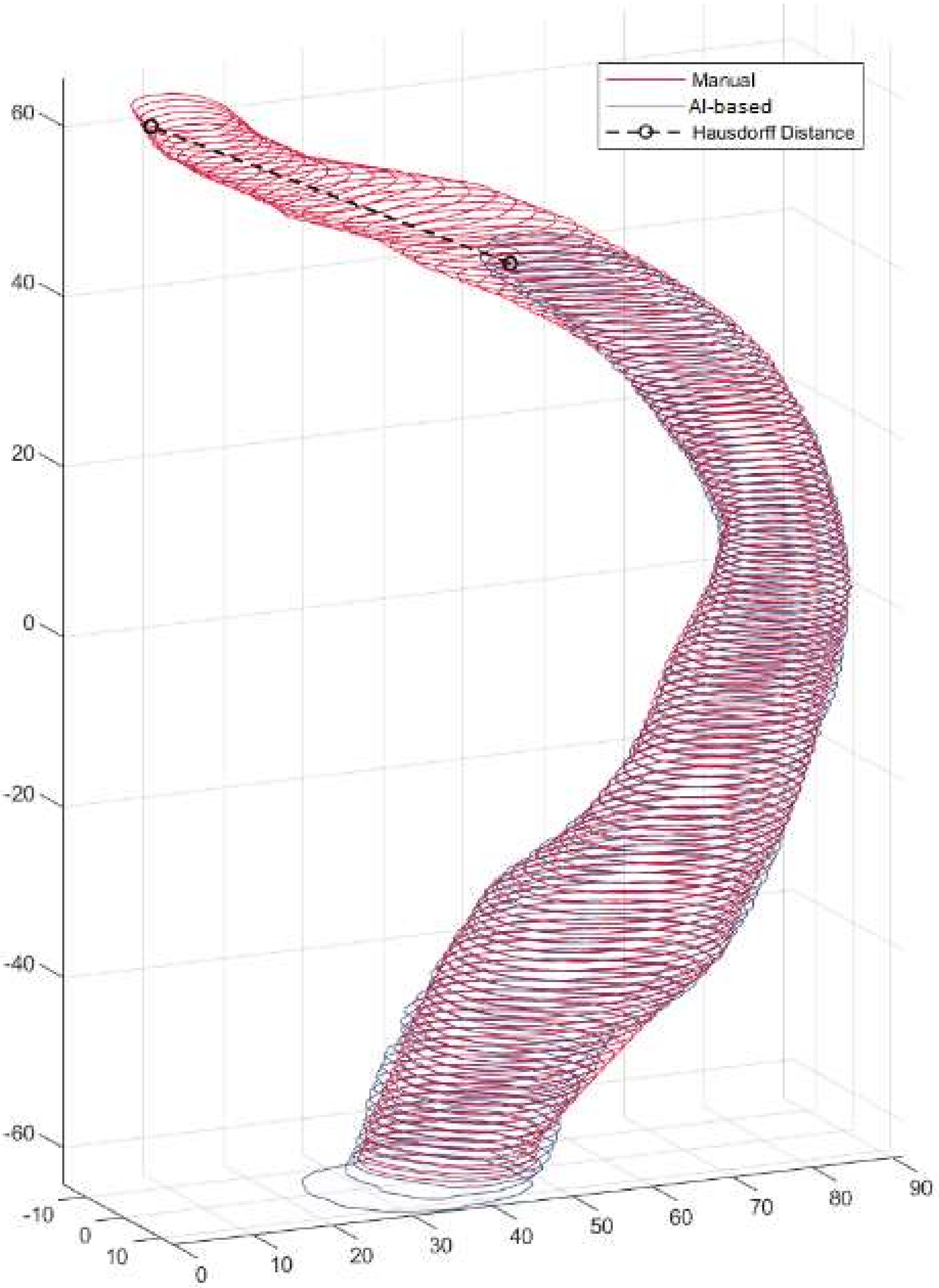

Figure S2: Analysis of the rectum contour for patient No. 18, showing a difference between the manual (red) and AI-based annotation (blue) in the cranial border.
